# The RhoA GEF Syx Is a Target of Rnd3 and Regulated via a Raf1-Like Ubiquitin-Related Domain

**DOI:** 10.1371/journal.pone.0012409

**Published:** 2010-08-25

**Authors:** Liuh Ling Goh, Ed Manser

**Affiliations:** 1 Rho GTPases in Stem Cells (RGS) Group, Institute of Medical Biology (IMB), Singapore, Singapore; 2 Small G-Protein Signaling and Kinases (sGSK) Group, Institute of Molecular and Cell Biology (IMCB), Neuroscience Research Partnership, Singapore, Singapore; University of Chicago, United States of America

## Abstract

**Background:**

Rnd3 (RhoE) protein belongs to the unique branch of Rho family GTPases that has low intrinsic GTPase activity and consequently remains constitutively active [1], [2]. The current consensus is that Rnd1 and Rnd3 function as important antagonists of RhoA signaling primarily by activating the ubiquitous p190 RhoGAP [3], but not by inhibiting the ROCK family kinases.

**Methodology/Principal Findings:**

Rnd3 is abundant in mouse embryonic stem (mES) cells and in an unbiased two-step affinity purification screen we identified a new Rnd3 target, termed synectin-binding RhoA exchange factor (Syx), by mass spectrometry. The Syx interaction with Rnd3 does not occur through the Syx DH domain but utilizes a region similar to the classic Raf1 Ras-binding domain (RBD), and most closely related to those in RGS12 and RGS14. We show that Syx behaves as a genuine effector of Rnd3 (and perhaps Rnd1), with binding characteristics similar to p190-RhoGAP. Morpholino-oligonucleotide knockdown of Syx in zebrafish at the one cell stage resulted in embryos with shortened anterior-posterior body axis: this phenotype was effectively rescued by introducing mouse Syx1b mRNA. A Rnd3-binding defective mutant of Syx1b mutated in the RBD (E164A/R165D) was more potent in rescuing the embryonic defects than wild-type Syx1b, showing that Rnd3 negatively regulates Syx activity *in vivo*.

**Conclusions/Significance:**

This study uncovers a well defined Rnd3 effector Syx which is widely expressed and directly impacts RhoA activation. Experiments conducted *in vivo* indicate that Rnd3 negatively regulates Syx, and that as a RhoA-GEF it plays a key role in early embryonic cell shape changes. Thus a connection to signaling via the planar cell polarity pathway is suggested.

## Introduction

Activation of most Rho family GTP binding proteins requires GDP-GTP exchange catalyzed by various guanine nucleotide exchange factors (GEFs) [4]. The GEFs of the Dbl family are Rho-specific exchange factors characterized by a catalytic Dbl-homology (DH) domain [5]. The Rnd proteins are unusual as they do not behave like conventional Rho proteins in requiring activation. Their low intrinsic GTPase activity, means they are predominantly in a GTP-bound state [1], [2], [6]: consequently Rnd proteins are regulated by altering protein levels or by post-translational modifications such as phosphorylation [7]. Several studies have demonstrated that the expression of Rnd3 (RhoE) increases in response to several signals [8], [9], [10]. Rnd proteins were discovered as potent antagonists of RhoA signaling by Nobes et al. [11], based on the phenotypic effects of Rnd proteins in adherent cells. Since then a handful of mechanisms have emerged to explain this observation: the inhibition of ROCK1 by Rnd3 [12] is not consistent with the structure of the complex [13], and does not involve the ‘effector’ regions of Rnd3; activation of p190 RhoGAP [3] is credible, but few details have emerged in support of this mechanism; finally interaction of Rnd3 with Socius a protein of unknown function has also been reported [14]. Clearly understanding of how Rnd1/3 antagonizes RhoA is the key to understanding its role.

Our knowledge of the *in vivo* roles for Rnds has come from work conducted with the *Xenopus* homologue xRnd1 which was found to regulate morphogenetic movements by modulating cell adhesion in early embryos [15]. During our gene expression profiling studies in mouse embryonic stem (mES) cells, Rnd3 was uncovered as one of the Rho GTPases that is highly expressed. This is of interest since Rnd proteins function as agonists of RhoA signaling. An important role for RhoA-ROK (ROCK) signaling in modulating the balance between proliferation and differentiation in embryonic stem cells has been described [16]. RhoA is also implicated in tissue morphogenesis during early development as exemplified by its role in the non-canonical Wnt signaling to activate DAAM1 and ROK kinases [17], [18]. These considerations prompted us to investigate the role of Rnd3 in the developmental context using mES cells and zebrafish embryos.

We started by using combined tandem affinity purification (TAP) and mass-spectrometry to identify proteins that bind Rnd3. We identified a single species, the synectin-binding RhoA exchange factor (Syx). This protein also known as GEF720/PLEKHG5/TECH is a Dbl-like protein most closely related to MyoGEF and p115-RhoGEF which are well characterized RhoA GEFs. Syx also acts on RhoA and is potentially involved in the control of neuronal cell differentiation [19], [20], [21]. We evaluated the structure-function relationship between Rnd3 and Syx and investigated the role of Syx during embryonic development in zebrafish. Contrary to published data we found that Syx is essential for early development of the embryonic anterior-posterior axis. A Rnd3-binding defective mutant of Syx is hyperactive, indicating Rnd3 functions to inhibit Syx *in vivo*. Thus Syx is a candidate GEF downstream of the Wnt-PCP pathway that connects Wnt to RhoA activation and suggests interplay among Syx, RhoA, ROK and Rnd3 in regulating convergent extension (CE) movements during Zebrafish gastrulation.

## Results

### Rnd3 co-purifies with a RhoA guanine exchange factor Syx

**Figure 1:**
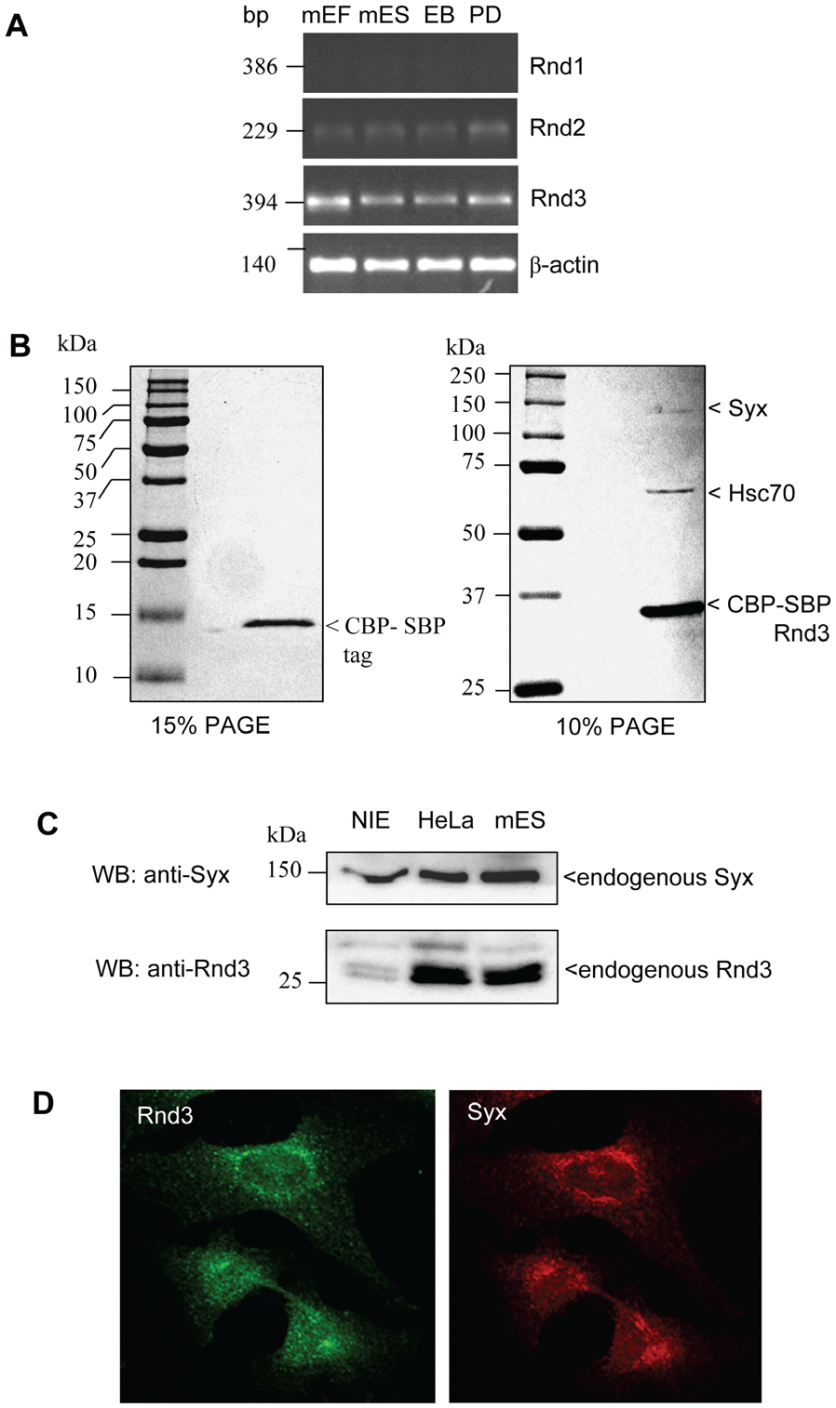
Rnd3 associates with Syx in mouse embryonic stem cells. (A) Expression analysis of Rnds evaluated by RT-PCR from mES cells, mouse embryonic fibroblast (mEF) cells, embryoid bodies (EB) or plated differentiated mES cells (PD). (B) Identification of Rnd3-associated proteins. Rnd3 TAP-tagged proteins from transiently transfected mES cells using CBP-SBP vector (see methods). The purified proteins by coomassie staining is shown with identification by LC-MS analysis. (C) Expression levels of Rnd3 and Syx in NIE-115, HeLa and mES cells. Cell lysates were resolved by SDS-PAGE and immunoblotted with anti-Rnd3 and anti-Syx. (D) Endogenous localizations of Rnd3 and Syx in HeLa cells immuno-labeled with anti-Syx and Rnd3 antibodies. Rnd3 and Syx are both localized near the perinuclear region. Bar = 10uM.

The role of RhoA-ROK signaling in embryonic stem cells is of some interest [16], [22], [23]. We therefore sought to investigate the expression and function of a known RhoA antagonist, Rnd3 in mES cells. Rnd1 and Rnd3 proteins down-regulate RhoA-GTP levels in a number of cell types [3], [12]. We investigated mRNA levels in undifferentiated and differentiated mES cells by RT-PCR: Rnd3 exhibited the highest expression level while Rnd1 expression was undetectable and Rnd2 expression was low (Figure 1A).

To assess if Rnd3 might have novel protein targets or modulators in mES cells we sought to identify these by affinity co-precipitation from mES cell lysates. Because Rnd proteins lack intrinsic hydrolysis [1], [6], one does not need to generate ‘constitutive active’ mutants to assess effector interaction. Wild-type (WT) Rnd3 was N-terminally tagged with the triple Calmodulin binding peptide (CBP), Streptavidin binding peptide (SBP) and Flag peptide. Tandem affinity purification was performed after transfection and expression of Rnd3 for 24 h in mES cells. Two co-purified proteins in the eluate of Rnd3 but not the vector control were observed by SDS-PAGE (Figure 1B). After in-gel digestion and tryptic digest LC-mass spectrometry, these matched to murine heat shock cognate protein (Hsc70, NP_112442) and synectin-binding RhoA exchange factor (Syx, AAU04953) respectively.

We sought to assess the localization of endogenous Rnd3 and Syx in adherent cells (which give better spatial resolution of the cytosolic compartment than ES cells). A number of cell lines were screened for the expressions of Syx and Rnd3 by western blotting. The affinity purified anti-Rnd3 and Syx antibodies (see methods section) detected both these proteins in lysates of human HeLa, mouse neuroblastoma NIE-115 and mES E14 cells, though the levels of Rnd3 protein is much lower in NIE115 (Figure 1C). Indirect immuno-fluorescence analysis showed that endogenous Rnd3 and Syx were not predominantly on the plasma membrane but rather in a perinuclear location. Both Rnd3 and Syx stainings were enriched in this perinuclear compartment and did not translocate when cells were switched from serum-free to serum containing media (Figure 1D).

**Figure 2:**
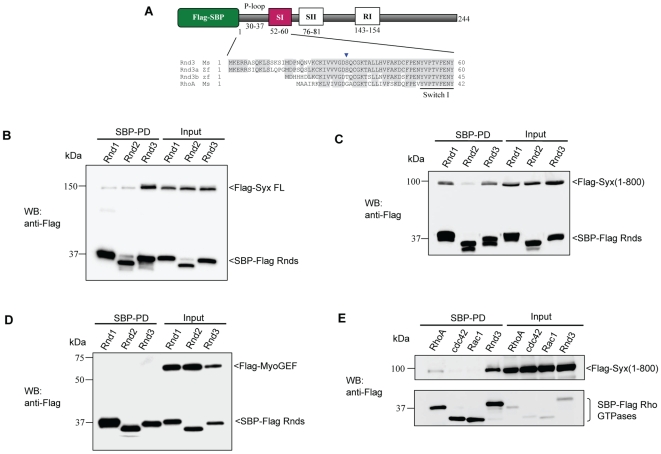
Analysis of the Rnd3-Syx interaction. (A) Schematic representation of Flag-SBP tagged Rnd3. The motifs of Rho family proteins are indicated: P-loop, the switch I and II (SI and SII), and Rho insert (RI) region. Flag (FL) and streptavidin binding peptide (SBP) tags are indicated. Sequence alignment of Rnd3 proteins showed that the N-terminal of zebrafish Rnd3a resembles more closely to mouse Rnd3. Identical residues are shaded in grey. Blue arrowhead denotes G14 in RhoA. Residue effector region is underlined. The accession numbers of mouse Rnd3 (NP083086), zebrafish Rnd3a (BC059452), Rnd3b (BC076009) and mouse RhoA (NP058082) are in brackets. (B and C) Characterization of Syx binding to Rnd isoforms. 293T cells were co-transfected with expression vectors encoding SBP-Flag Rnd1, Rnd2 or Rnd3 and full-length Syx or Syx(1–800) as indicated. Lysates were subjected to SBP pulldown and immunoblotting. (D) Rnd3 does not interact with MyoGEF. 293T cells were co-transfected as for (B) with Flag-MyoGEF and assessed for interaction. (E) Selective association of Syx with Rnd3. Co-expression of SBP-Flag RhoA(GV14), Cdc42(G12V), Rac1(G12V) or Rnd3 with Syx(1–800) and immunoblotted for Flag epitope.

We tested the ability of Syx to interact with the two other mouse Rnd isoforms using streptavidin pulldowns, which avoid artifacts associated with antibody chains following standard immuno-precipitation. The SBP-Rnd fusion proteins (Figure 2A) also contain a Flag peptide sequence which allows us to assess the relative levels the ‘bait’ and Flag tagged protein partners in the pull-down (SBP-PD). For clarity and honesty with respect to the efficacy of pull-down, all components are shown in a single panels. In most cases the proteins could be detected by coomassie staining of the PVDF blots (data not shown). Syx was recovered with Rnd3 at a reasonable yield, but little binding of full-length Syx to Rnd1 or Rnd2 was evident (Figure 2B). By contrast Syx(1–800), which is likely to be relieved from some auto-inhibitory constraint [21], bound both Rnd1 and Rnd3 (Figure 2C). Since full-length Syx could not be purified from bacteria, further *in vitro* characterization of the activity of this protein was not possible. Syx is a RhoGEF most closely related to MyoGEF, exhibiting 64% and 75% similarity in their DH and PH domains respectively. No interaction was observed between MyoGEF and the Rnd proteins (Figure 2D), suggesting that Rnd binding resides within regions that are unique to Syx. We also confirmed that Syx is a RhoA binding GEF [19], [21] but does not appreciably interact with Cdc42 or Rac1 (Figure 2E).

### Identification of the Syx region that binds Rnd3

**Figure 3:**
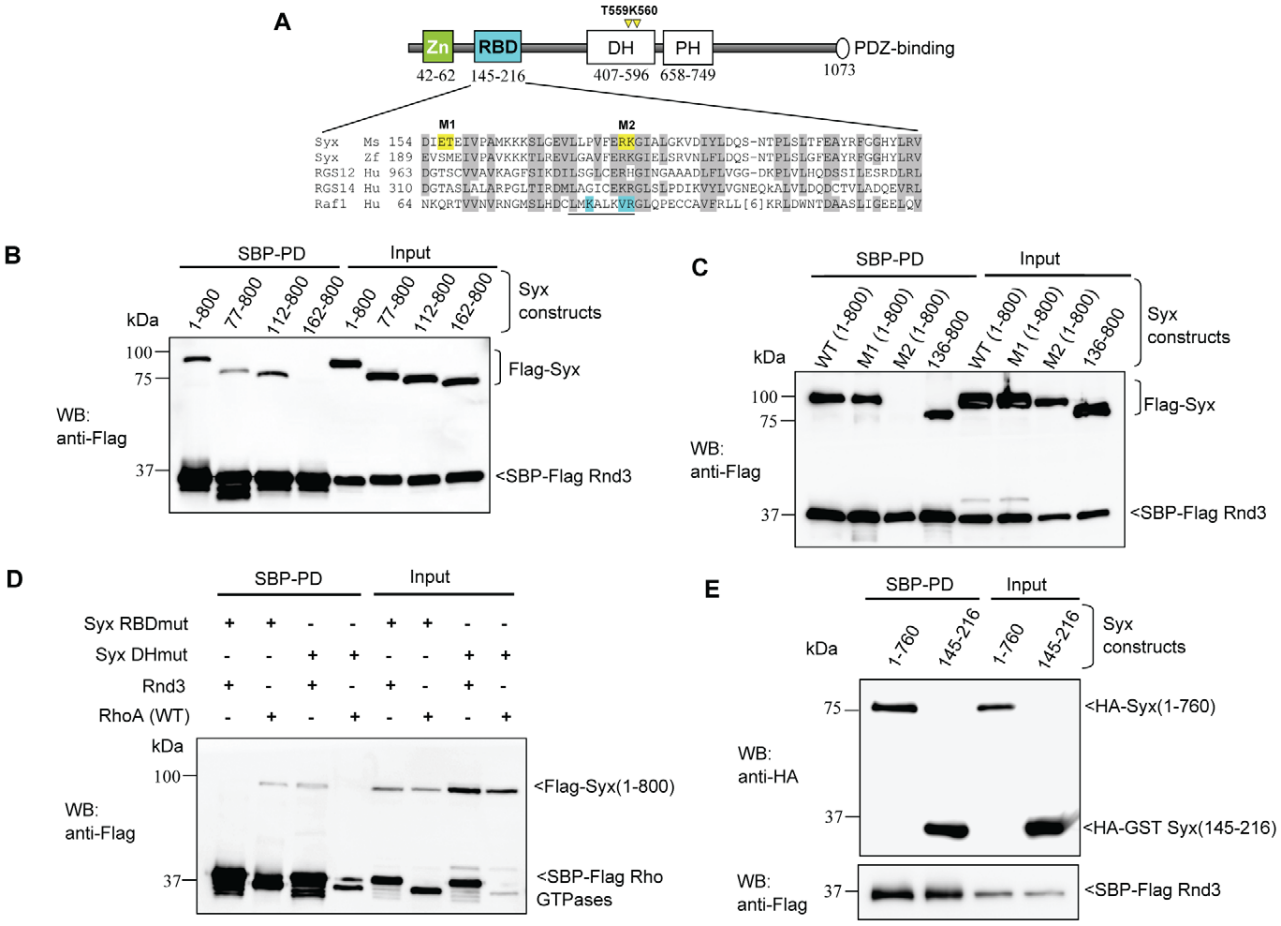
Characterization of the interaction between Rnd3 and Syx. (A) Schematic of domains identified in Syx: including zinc-finger, Rnd3-binding (in cyan), Dbl homology (DH), pleckstrin homology (PH) and PDZ-binding motif. Sequence alignment of Raf1-like RBDs in mouse Syx (AAU04953), zebrafish Syx (XM_686228.1), human RGS12 (NP_002917), RGS14 (NP_006471) and Raf1 (AAA60247). Conserved residues are shaded in grey; a key helix is underlined; amino acid side chains contacting Ras are in cyan and residues mutated in this study are in yellow. The catalytic residues T559/K560 in Syx DH domain are denoted by yellow arrowheads. (B) Rnd3 interacts with the N-terminal of Syx. 293T cells were co-transfected with SBP-Flag Rnd3 and indicated Flag-Syx constructs. The purified protein complex was immunoblotted to visualize bound Syx. (C) R178 and K179 of Syx are critical Rnd3 binding residues. 293T cells were co-transfected as for (B) with the indicated constructs and assessed for interaction. M1 and M2 refer to the mutant constructs, E156AT157A and R178EK179D. (D) Rnd3 and RhoA (WT) interact with Syx via the RBD and DH domain, respectively. The mutant constructs, R178EK179D and T559EK560E are represented by Syx RBDmut and Syx DHmut, respectively. (E) Rnd3 interacts with Syx(145–216).

The Syx protein has a number of domains that show homology to other proteins including the DH domain (407–596) that accelerates the exchange of nucleotides on Rho family GTPases, and the PH domain (658–749), a PDZ-binding domain at the C-terminus, and a putative zinc finger region with little sequence similarity outside the cysteine-histidine residues (Figure 3A). Various Syx truncations were expressed and tested for Rnd3 binding in 293T cells lysates by SBP pull-down. Syx(1–800), Syx(1–657) and Syx(1–393) lacking the DH/PH domain all readily bound to Rnd3 while Syx (381–1033) did not show any interaction (Figure S1). Thus the N-terminal of Syx is necessary and sufficient for its interaction with Rnd3. This was surprising given that only the DH domain of this protein would be expected to interact with Rho proteins. We found that N-terminal truncations Syx(77–800) and Syx(112–800) could bind to Rnd3 while Syx(162–800) could not (Figure 3B), implicating residues downstream of amino acid 112 in binding, a region that lies outside the zinc finger domain.

We determined visually that a 63 amino acid region (154–216) has significant homology with the putative Ras binding domains (RBD) of RGS12 and RGS14 (Figure 3A). Based on the crystal structure of Rap1a complexed with the RBD of Raf1 [24], the side chains of T68, K84 and R89 of Raf1 (marked in blue) are known to directly contact K-Ras. We then created two mutants, Syx(1–800) E156A/T157A (exposed side-chains not directly involved in effector binding) and R178E/K179D (equivalent to the Raf1 K84/R89 side-chains involved with Ras binding). In Figure 3C we see that Syx(136–800) fragment lacking sequences N-terminal to the putative RBD bound well to Rnd3, as did the Syx(1–800) E156A/T157A; it was clear however that the R178E/K179D substitutions prevented Syx binding to Rnd3. These support the notion that Syx RBD extends from 145–216 and is involved in Rnd3 binding. This Rnd3 interaction site is quite distinct from the RhoA exchange domain. The binding of RhoA to the Syx DH domain (Figure 3D) was confirmed by the Syx T559E/K560E substitutions in the RhoGEF DH domain which abolishe RhoA but not Rnd3 binding. We also confirmed that this RBD-like motif alone Syx(145–216) binds well to Rnd3 (Figure 3E), demonstrating that the Syx ubiquitin-fold RBD is involved in binding Rnd3 in a manner similar to the effector Raf1 with Ras. This contrasts with the Rac1/Rnd3/RhoD binding domain of plexin B1 which is ubiquitin-like in nature but binds to these GTPases with a completely different ‘face’ [25]. It has not escaped our notice that other ‘orphan’ RBDs that have been tested and do not bind Ras-like GTPases [26] might well be Rnd or Rho family targets. These results will be presented elsewhere.

### Syx is essential for normal gastrulation in zebrafish embryos

**Figure 4:**
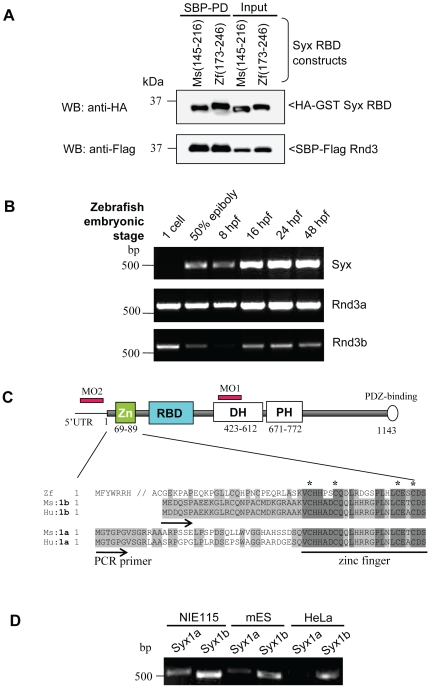
Zebrafish Syx is closely related to mammalian Syx1b and expressed from epibolic stages. (A) Zebrafish Syx interacts with mouse Rnd3 via its RBD domain (residues 173–246). (B) Expression of Syx and Rnds mRNA. RT-PCR analysis showing mRNA transcript profiles of zebrafish Syx, Rnd3a and Rnd3b at different developmental stages indicated. Primers to Syx (nucleotide 2640–3120) (XM001923778), Rnd3a (nucleotide 1–732) (BC059452) and Rnd3b (nucleotide 1–672) (BC076009) were used. (C) Schematic representation of zebrafish Syx, including the conserved RBD, DH/PH domains and PDZ-binding motif. The N-terminal region of zebrafish Syx resembles more closely to mammalian Syx1b than the published Syx1a. Conserved residues are in light grey and identical residues in dark grey. The zinc finger motif is underlined and the conserved cysteine residues marked (*). Database numbers for zebrafish Syx (XM_686228.1), mouse Syx1b (B1AS67), human Syx1b (094827), mouse Syx1a (AAU04953) and human Syx1a (AK299523) are in brackets. (D) RT-PCR revelas that Syx 1b is more highly expressed in mouse NIE115, ES and human HeLa cells. RT-PCR from alternative start sites to a common downstream primer (nucleotide 495).

The inhibitory role of Rnds on RhoA signaling is well documented [3], [12], [27], [28], [29]. Given the complex interplay between Rnd3, RhoA and p190 RhoGAP in cell based assays, we sought to use the developmental requirement for Syx [30], [31] as a means of assessing Rnd3's role in binding to Syx. While zebrafish and mouse Rnd3 proteins are highly conserved (∼90% similarity), zebrafish Syx shares moderate similarity (∼60%) with its mouse orthologue. We first validated the physiological relevance of Rnd3/Syx interaction in zebrafish by showing that zebrafish Syx RBD(173–246) is capable of binding to Rnd3, to the same extent as mouse Syx RBD(145–216) (Figure 4A). Next, the potential role of Rnd proteins in zebrafish development was assessed by investigating their temporal expression. The two Rnd3 paralogues, Rnd3a and Rnd3b are expressed maternally (Figure 4B), but Rnd3a is likely dominant. Zebrafish Rnd3a closely resembles human and mouse Rnd3 while the N-terminal extension is absent in Rnd3b (alignment in Figure 2A). Syx is expressed at the onset of gastrulation (50% epiboly), and mRNA levels increase during embryogenesis from 16 hpf to 48 hpf. Published *in situ* hybridization indicated that Syx is uniformly expressed in the embryo during gastrulation [30]. Zebrafish WGEF (RhoGEF19), a RhoA GEF implicated in *Xenopus* gastrulation [18] could not be detected at this stage (not shown).

**Figure 5:**
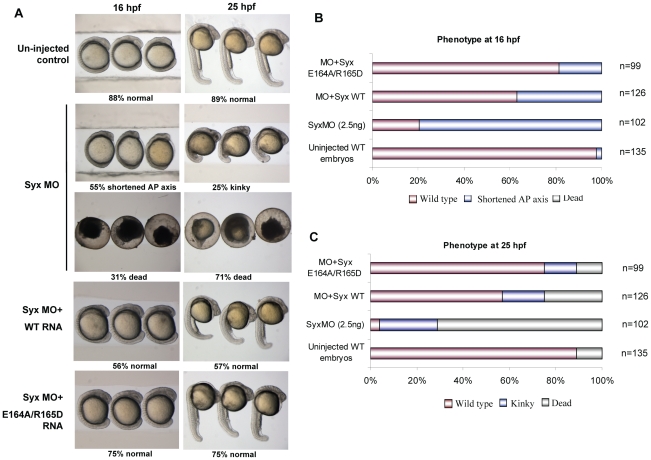
Loss of Syx causes gastrulation defects and death that can be rescued by mouse Syx1b mRNA. (A) Typical phenotypes observed in zebrafish embryos at 16 and 25 hpf. At 16 hpf, Syx depleted embryos displayed shortened AP axis, indicative of gastrulation defects. Co-injection of Syx MO with mouse Syx1b mRNA reduced both death and gastrulation defects. Co-injection of Syx E164A/R165D mRNA was more effective than wildtype (WT) Syx. At 25 hpf, Syx morphants had shorter body length and kinky tail (though most died). Rescued phenotypes of WT and E164A/R165D at 25 hpf resembled un-injected controls. (B and C) Quantification of embryos (n is the total number scored) showing penetrance of Syx morphants and rescue through co-injection of Syx1b WT or E164A/R165D RNA at 16 and 25 hpf.

Two Syx-specific morpholino-oligonucleotides (MOs), which have been well characterized previously [30], can efficiently knockdown or cause mis-splicing of zebrafish Syx transcripts. The MO1 targeting the splicing junction at the functional DH domain of Syx (Figure 4C) was highly penetrant at 2.5 ng, producing pronounced shortening of the anterior-posterior (AP) body axis (Figure 5A and 5B), although these exhibited normal epibolic closure (4–8 hpf). The shortened axis of Syx morphants resemble convergence and extension (CE) defects seen with knockdown of *wnt11* [32] or RhoA [33]. Most embryos (∼70%) did not survive to 25 hpf (Figure 5C). The few surviving morphants were characterized by short and kinky tails (Figure 5A). The off-target effects of many MOs in zebrafish involve p53 mediated apoptosis [34]: however the Syx morphant phenotype was maintained in the presence of 4.5 ng p53 MO (Figure S2). Thus the early phenotype associated with the Syx is consistent with it playing an important role in vertebrate gastrulation. It is notable that a Syx-like protein is found also in primitive lancelet chordates (Figure S3).

### Mammalian Syx can rescue zebrafish gastrulation defects

The zebrafish Syx transcript was divergent from published mammalian Syx proteins around the translational start site, but we found human and mouse EST variants more closely resembling the zebrafish sequence in this region (Figure 4C). The published transcripts (designate here as Syx1a) are significantly less abundant in both mouse (NIE115, mES) and human (HeLa) cell lines than our new Syx1b (Figure 4D). Unlike ‘rescue’ experiments in mammalian cells treated with siRNA, one can carefully titrate the amount of synthetic mRNA microinjected into zebrafish embryos. Full-length mouse capped Syx1b mRNA (100 pg) when co-injected with Syx MO was able to efficiently rescue the phenotypes associated with the loss of zebrafish Syx (Figure 5A and 5B). These experiments were carefully controlled for injection efficiency (see methods). Remarkably at 25 hpf, 57% of the rescued embryos were morphologically normal, versus 4% for Syx morphants (Figure 5C). The results demonstrated that mouse Syx1b can efficiently and functionally replace zebrafish Syx in developing embryos, indicating evolutionarily conserved protein function; and that the MO effects are specific to Syx.

Rnd3 function in zebrafish is complicated by the presence of multiple isoforms. Morpholino (5 ng) targeted against Rnd3a was lethal at 48 hpf though compensatory contribution from Rnd3b is possible. To address the role of Rnd3 in the regulation of Syx during gastrulation, we compared the rescue efficiency of Rnd3-binding defective Syx1b(E164A/R165D) versus Syx1b WT (Figure 5). At 25 hpf, co-injected Syx1b WT yielded ∼57% normal while this increased to ∼75% for the mutant Syx (Figure 5C), which in our experience is as high as can be achieved in these type of manipulations. This data indicates that interaction with Rnd3 is not required for Syx function, and is consistent with a negative regulatory role by Rnd3. Thus Rnd3 down-regulation of Syx would antagonize RhoA activation, as have been observed in cell culture [6], [35], [36].

## Discussion

### Syx as a genuine effector of Rnd3

This study started with a proteomic approach to identify Rnd3 targets in mES E14 cells and revealed Syx to be relatively abundant in mES, neuroblastomas and Hela cells. Although Syx has been described as a neuronally enriched protein [19], [21] it may well participate in Rnd3 signaling in other contexts and is widely expressed in non-neuronal cells in culture. That Rnd3 binds to a region in the N-terminal of Syx sharing sequence similarity with the Raf-1 RBD is particularly interesting. Such RBDs are found in a wide variety of signaling proteins and many are reported not to bind to the Ras-subfamily (cf. Ras, Ral, Rit) [26]. Based on our Syx RBD mutagenesis analysis (Figure 3), Syx likely binds Rnd3 in a manner resembling the Rap1A-Raf1 complex [24]. Other RhoGEFs have been found with classical RBDs. For example Tiam-1, a Rac specific RhoGEF that is activated by Ras.GTP through its RBD [37] and SOS, the key Ras-GEF that is also regulated by Ras.GTP as well as coupled to Rac1 activation [38]. The DH domain is not involved in Rnd3 binding (Figure 3E).

**Figure 6:**
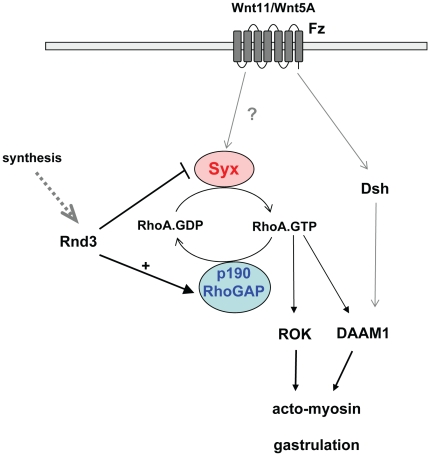
A model of the interplay between RhoA and Rnd3 pathways. Syx-mediated RhoA signaling regulates CE movements in zebrafish via ROK and DAAM1. Rnd3 can down-regulate RhoA function by activating p190 RhoGAP or inhibiting Syx.

Rnd3 can down-regulate RhoA.GTP levels by activating p190RhoGAP via an as yet poorly defined binding domain [3]; a complementary mechanism would be to inhibit RhoGEFs such as Syx (Figure 6). The Rnd3-ROKβ interaction that leads to Rnd3 phosphorylation is independent of its switch I/effector region of Rnd3, and does not contribute to Rnd3-induced loss of actin stress fibers [13].

### Syx, a Rho exchange factor with roles in early development

We describe here additional features of the Syx protein that have not been described previously, including a zinc finger and the ubiquitin-like RBD that is responsible for binding to Rnd3. C-terminal deletion of Syx appears to increase Rnd3 binding, indicating an intramolecular N- to C-terminal auto-inhibition that might be relieved by Rnd3 association as seen for other RhoGEFs [39], [40].

In zebrafish and *Xenopus*, inhibition of RhoA signaling caused gastrulation defects, resulting in embryo with a short body axis or curly tail [32], [33], [41], [42]. Knockdown of Syx causes embryonic defects similar to those of RhoA, ROK or Wnt morphants. Previously it was reported that Syx loss-of function in the zebrafish causes defects in angiogenesis based on the observation that Syx is associated with Angiomotin, a scaffold protein implicated in vascular patterning and required for endothelial cell polarization and migration during embryogenesis [30], [31]. While we make no comment on these previous reports, in our hands Syx is required much earlier in development, and at lower MO levels general delays in development rather than specifically vascularisation occur. We consider it likely that Syx acts downstream in the non-canonical Wnt pathways that control early morphogenesis through RhoA effectors such as DAAM1 [17], [18]. There is compelling evidence that RhoA cooperates with Wnt/Fz to promote important aspects of CE [17]. In zebrafish, expression of RhoA rescued Wnt11 mutant to mediate CE movements [41]. Less is known about the immediate upstream regulator of RhoA during gastrulation. To date only WGEF, a Rho-specific GEF in Xenopus has been shown to be essential in this early phase of development [18]. However, the WGEF homologue is not present in the zebrafish. Our results provide the first evidence that Syx is a RhoGEF required for CE in zebrafish in which Rnd3 plays an antagonistic role. Because of the presence of multiple Rnd3 genes in zebrafish, further studies require careful analysis of temporal and spatial expression of the proteins.

In conclusions, biochemical characterization of Rnd3 targets in mES cells by TAP tagging indicated Syx as a relatively abundant protein partner under our conditions. We showed that Rnd3 interacts via a region that resembles a classic Ras-binding domain, and that mutations at the putative Syx-Rnd3 interface disrupt this. Using this system, we uncovered the role of Syx during the period of gastrulation when it is already established that RhoA plays key roles in signaling downstream of Wnt.

## Materials and Methods

### Cell culture and transfection

Mouse ES cell line E14 (ATCC CRL-1821) was obtained from Genome Institute of Singapore and cultured on tissue culture plates (Corning) coated with 0.5% porcine gelatin (Sigma). HEK 293T (ATCC CRL-11268) and HeLa cells (ATCC CCL-2) were purchased from American Type Culture Collection. Transient transfections were carried out with Lipofectamine 2000 (Invitrogen) for mouse ES cells or Fugene 6 (Roche) for HEK 293T and HeLa cells, according to the manufacturer's instructions.

### Plasmid constructions

Mouse full length Syx in pcDNA3.1 was a generous gift from Arie Horowitz (Dartmouth Medical School). The full length and various truncated constructs of Syx were obtained by PCR amplification and subcloned into N-terminus fusion versions of Flag tagged pXJ40 vectors. Mouse MyoGEF was purchased from Open Biosystems (Clone ID 40034695) and subcloned into HA-GST tagged pXJ40 vector. Rnd1, Rnd2, Rnd3, RhoA and ROKβ were cloned from mES cell line E14 by reverse transcription-PCR (RT-PCR). Syx1b was cloned from neuroblastoma NIE-115 (ATCC CRL-2263) cells by RT-PCR. Mouse RhoA, Cdc42 and Rac were cloned into N-terminus fusion versions of SBP-Flag tagged pXJ40 vectors. To generate the construct for tandem affinity purification, SBP and CBP were fused in frame to the Flag tag of pXJ40 using BamHI/HindIII sites. Mutants of Rnd3 (T37N, T55A and C241S), RhoA (G14V), Syx (E156A/T157A and R178E/K179D) and Syx1b (E164A/R165D) were generated by PCR-mediated mutagenesis using the QuikChange II protocol (Stratagene) and sequenced.

### Tandem affinity purification

Tandem affinity purification was performed using mES cells lysates with overexpressed CBP and SBP-tagged Rnd3 expression vector. Briefly, 25 T75 cm_2_-plates were lysed in buffer containing 10 mM Tris/HCl pH 8.0, 250 mM NaCl, 0.1% Nonidet P-40, protease inhibitor cocktail (Complete Mini EDTA-free; Roche) and 2 mM sodium-orthovanadate. Binding of cell lysates to streptavidin resin (50% slurry) (Amersham Pharmacia Biotech) was performed at 4°C for 2h. Extensive washing with streptavidin binding buffer (SBB) was followed by 30 min incubation with 2 mM biotin at 4°C. The eluate was applied to calmodulin affinity resin (Stratagene). After 2h incubation at 4°C, the beads were washed repeatedly with calmodulin binding buffer. Finally, calmodulin bound protein complexes were recovered by 30 min elution with 5 mM EGTA. TAP-associated proteins were separated by SDS-PAGE, coommassie-stained, and excised for analysis by LC/MS.

### Streptavidin pulldown

Cells were transiently transfected with the indicated combination of plasmids and harvested at 24 h. Cells were washed in phosphate-buffered saline (PBS), lysed as described above, and centrifuged at 13 000 rpm for 10 min at 4°C. The supernatants were incubated with 10 µl of streptavidin beads for 1h at 4°C; washed 3 times and eluted with 2 mM biotin. The eluate containing the interacting complex was separated by SDS-PAGE, transferred to PVDF and immunoblotted with the indicated antibodies.

### Immunofluorescence Microscopy

HeLa cells seeded onto 13 mm glass coverslips were washed with cold PBS and fixed with 4% paraformaldehyde for 30 min at 4°C. Fixed cells were permeabilization with 0.1% saponin for 15 min. Following this, the coverslips were incubated with primary antibodies at room temperature for 1h. Coverslips were then washed three times in 0.1% saponin-containing PBS before incubation with secondary fluorescent antibodies (Molecular Probes) coupled with Alexa 488 or 564. Images were viewed with Olympus laser scanning microscope (FV-1000). All images were visualized with 60× objective lens.

### Antibodies

The following antibodies were used: anti-FLAG (M2), anti-HA (HA-7), and anti-Rnd3 (Upstate). Rabbit polyclonal antibody to mouse Syx was raised (Genemed Synthesis, Inc) and affinity purified using a peptide (QHRKLTLAQLYRIRTT) corresponding to residues 1046–1061. For western blotting, the primary antibodies were detected using horseradish peroxidase-conjugated secondary antibodies (BioRad) with Amersham ECL or SuperSignal West Dura detection reagents.

### Morpholino injections and rescue experiments

The zebrafish Syx gene (XM_686228.1) was knockdown using morpholino (MO) oligonucleotides described previously [30]. One is designed against the 5′UTR sequence of Syx (5′CATGCCTTCGCCAATAGAACATCGT3′) and the other against a splicing junction at the DH domain (5′AGCTGTTTCTGTGTGGCCTGCTGA3′) which leads to complete loss of normal splicing. MO against p53 (5′GCGCCATTGCTTTGCAAGAATTG3′) was used to test for off target effect. All MOs were purchased from Gene tools LLC and reconstituted in nuclease free water to a stock of 10 mg/ml. The MOs were injected, with Texas-red dextran as a tracking dye, into each embryo at 1 cell stage using the IM300 microinjector (Narshige). After incubation at 28°C for 6h, the embryos were manually selected for quality of injection. Those with high or low level of fluorescent dye were rejected. The embryos were incubated at 24°C overnight and the developmental stage assessed from the controls. For rescue experiments, full-length mouse Syx1b was cloned into pXJ-vector and capped mRNA was synthesized using 1mM ribonucleoside triphosphate set with 0.5 mM m7G(5′)ppp(5′)G and 20U of T7 RNA polymerase (Roche). RNA was purified by phenol/chloroform treatment and precipitated with 4 M lithium chloride. Different concentrations of capped Syx1b RNA were titrated to obtain optimal rescue at a level where the RNA alone gave minimal or no defect prior rescue experiment. Rescue was then performed by co-injecting synthetic capped RNA at optimal concentration (100 pg) with Syx MO.

## Supporting Information

Figure S1 Rnd3 interacts with the N-terminal of Syx. 293T cells were co-transfected with SBP-Flag Rnd3 along with the indicated Flag-tagged Syx constructs. Cells were lysed 24 h post-transfection, and cleared lysates were subjected to SBP pulldown. The purified protein complex was analyzed by immunoblotting with anti-Flag antibody to visualize the associated proteins.
(0.12 MB TIF)

Figure S2 Syx morphant phenotype was maintained in the presence of p53 MO. Typical phenotypes observed in zebrafish embryos at 15 and 20 hpf. At 15 hpf, Syx depleted embryos displayed shortened AP axis. Co-injection of Syx MO (2.5 ng) with p53 MO (4.5 ng) did not reduce death and gastrulation defects. At 20 hpf, Syx morphants and those coinjected with p53 have shorter body length (though most died). Phenotypes of p53 MO injected embryos resembled un-injected controls.
(0.49 MB TIF)

Figure S3 Sequence alignment of Syx proteins. The N-terminal zinc finger and RBD motifs (underlined) of Syx proteins are conserved from primitive lancelet (Branchiostoma floridae, XP_002590453) to zebrafish (Danio rerio, XM_686228.1) and human (Homo sapiens, 094827) vertebrates. Identical residues are boxed in dark grey and conserved residues are in light grey. Red arrowheads indicate the RhoGEF domain.
(0.77 MB TIF)
