# Supplementary figures and images for: The RhoA GEF Syx Is a Target of Rnd3 and Regulated via a Raf1-Like Ubiquitin-Related Domain
Source: PLoS One. 2010 Aug 25;5(8):e12409. doi: 10.1371/journal.pone.0012409 (PMC2928299; doi:10.1371/journal.pone.0012409)

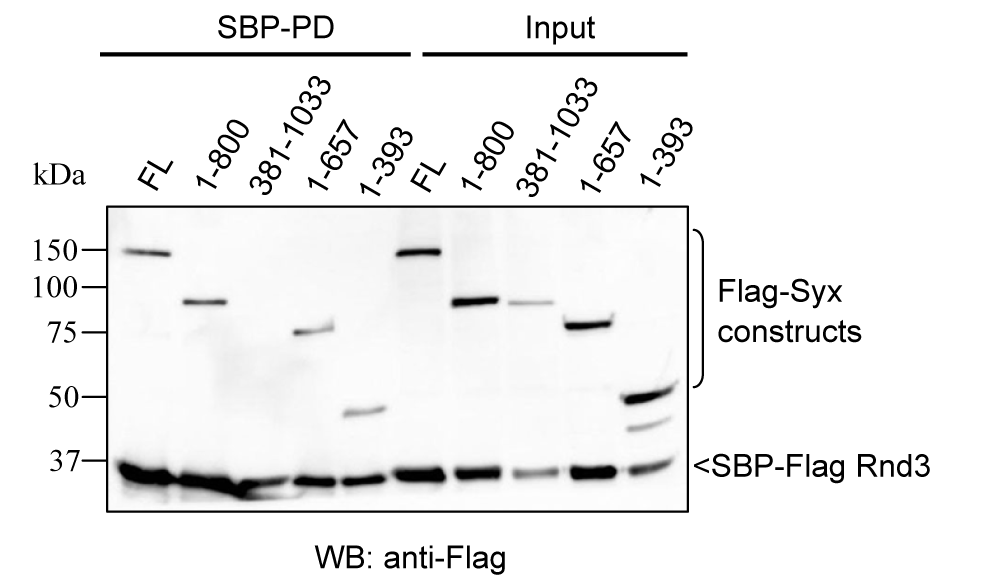

Supplement: Figure S1 — Rnd3 interacts with the N-terminal of Syx. 293T cells were co-transfected with SBP-Flag Rnd3 along with the indicated Flag-tagged Syx constructs. Cells were lysed 24 h post-transfection, and cleared lysates were subjected to SBP pulldown. The purified protein complex was analyzed by immunoblotting with anti-Flag antibody to visualize the associated proteins. (0.12 MB TIF) [file pone.0012409.s001.tif]

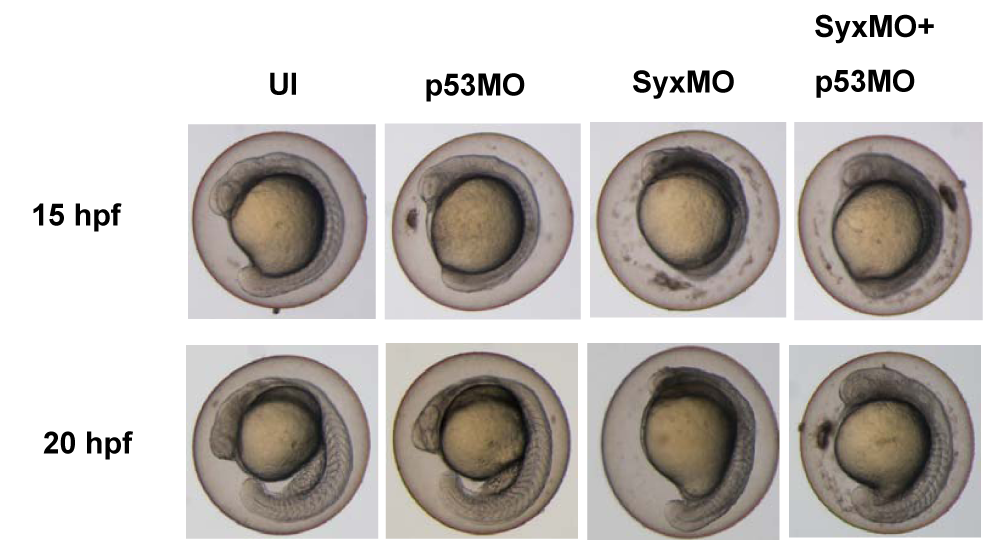

Supplement: Figure S2 — Syx morphant phenotype was maintained in the presence of p53 MO. Typical phenotypes observed in zebrafish embryos at 15 and 20 hpf. At 15 hpf, Syx depleted embryos displayed shortened AP axis. Co-injection of Syx MO (2.5 ng) with p53 MO (4.5 ng) did not reduce death and gastrulation defects. At 20 hpf, Syx morphants and those coinjected with p53 have shorter body length (though most died). Phenotypes of p53 MO injected embryos resembled un-injected controls. (0.49 MB TIF) [file pone.0012409.s002.tif]

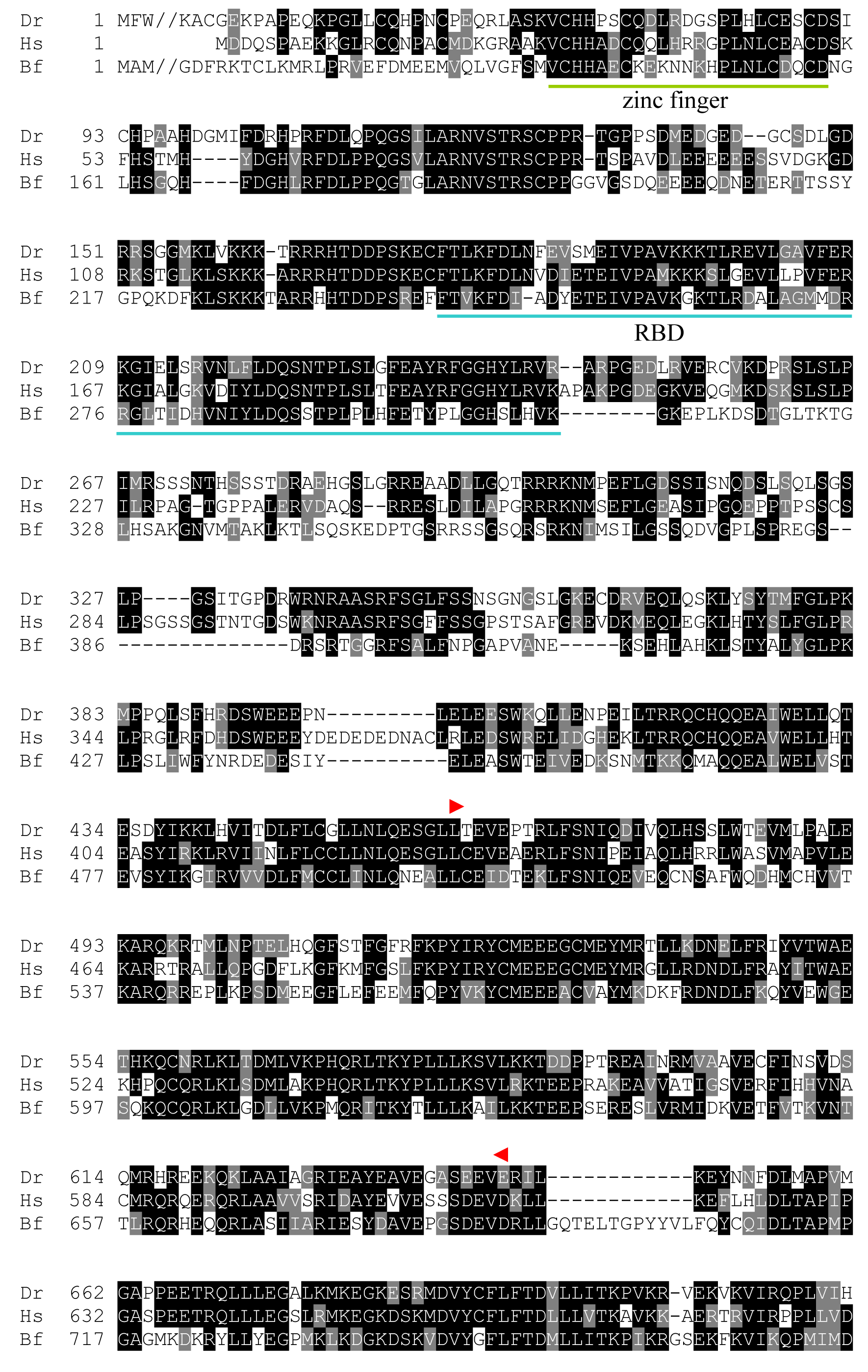

Supplement: Figure S3 — Sequence alignment of Syx proteins. The N-terminal zinc finger and RBD motifs (underlined) of Syx proteins are conserved from primitive lancelet (Branchiostoma floridae, XP_002590453) to zebrafish (Danio rerio, XM_686228.1) and human (Homo sapiens, 094827) vertebrates. Identical residues are boxed in dark grey and conserved residues are in light grey. Red arrowheads indicate the RhoGEF domain. (0.77 MB TIF) [file pone.0012409.s003.tif]
